# Investigating fear, depressive symptoms and coping mechanisms among Egyptian nursing students amidst the covid-19 pandemic: a cross-sectional study

**DOI:** 10.1186/s12912-024-02104-7

**Published:** 2024-07-08

**Authors:** Mona Metwally El-Sayed, Ghada Ahmed Ghazi, Manar Ahmed Kamal, Mahmoud Abdelwahab Khedr

**Affiliations:** 1 Psychiatric and Mental Health Nursing, Faculty of Nursing, Alexandria University, Alexandria, Egypt; 2 Faculty of Nursing, Psychiatric Nursing and Mental Health, Alexandria University, Alexandria, Egypt; 3 Faculty of Medicine, Banha University, Banha, Egypt

**Keywords:** Fear, Depressive Symptoms, Coping Mechanisms, Egyptian Nursing, COVID-19

## Abstract

**Background:**

Amidst the ongoing COVID-19 pandemic, nursing students' contribution to combating this global health crisis is becoming more significant. However, we need a more comprehensive understanding of the psychological impact of this crisis on these students. Therefore, this study holds immense importance as it offers valuable insights into the connection between COVID-19 pandemic fear, coping strategies, and depressive symptoms among nursing students.

**Methods:**

A cross-sectional survey was conducted to collect and analyze data from 319 undergraduate nursing students. They participated in the study by responding to an online questionnaire. The questionnaire consisted of the Fear of the Coronavirus Questionnaire, Brief Coping Orientation to Problems Experienced, and the Beck Depression Inventory-II.

**Results:**

The study revealed that 45.5% of the participants had a severe fear of COVID-19, 21.9% showed severe depressive symptoms, and 64.6% used moderate coping mechanisms. A positive correlation (*r* = 0.160, *p* = 0.01) was found between the fear of COVID-19 and depressive symptoms. Interestingly, this correlation turned negative (*r* = -0.074, *p* = 0.01) when adaptive coping strategies such as religion, acceptance, planning, positive reframing, instrumental support, emotional support, active coping, and humor were used. However, when participants resorted to maladaptive coping strategies like venting, self-distraction, self-blame, behavioral disengagement, denial, and substance abuse, the correlation between high fear levels and depressive symptoms became positive (*r* = 0.352, *p* = 0.01).

**Conclusion:**

The study demonstrated that the fear of COVID-19 and depressive symptoms among nursing students are significantly correlated. However, the nature of this correlation is influenced by the type of coping strategies employed. Adaptive coping mechanisms can mitigate the impact of fear on depressive symptoms. In contrast, maladaptive coping can exacerbate the relationship between fear and depressive symptoms. Therefore, promoting adaptive coping strategies could be a crucial approach to managing the psychological impact of the COVID-19 pandemic.

## Introduction

In 2020, the novel coronavirus, also known as COVID-19, was officially classified as a global pandemic by the World Health Organization (WHO) [1]. This respiratory virus spreads primarily through contact with an infected person and through respiratory droplets generated by coughing or sneezing, which can be inhaled or contaminated by hands and surfaces [1]. Due to the COVID-19 pandemic, there has been a significant rise in the number of infected cases globally, particularly among vulnerable groups at a higher risk of mortality. WHO documented a worldwide total of more than 520 million confirmed COVID-19 cases and 6.3 million fatalities. In Egypt, specifically, there were 513,944 confirmed cases of COVID-19 and 24,718 deaths caused by the virus [1]. This has created a feeling of uncertainty and anxiety on a global level. The uncertainty not only affects physical health but also has various effects on mental health, including sleep disorders, depression, and post-traumatic stress symptoms [2, 3].

Research worldwide has underscored the physical, mental, and psychological impacts of COVID-19. The recent virus surge has instilled widespread fear and stress among the general populace [4–6]. Students are particularly anxious about their health, safety, and education, which could negatively impact their learning, academic performance, and mental and psychological health [5]. To curb the spread of COVID-19, students had to adjust to new living conditions and use various online learning platforms [6]. They also grapple with other issues, such as significant worries about their health and that of their family members and financial concerns [7]. These sudden changes and challenges have been found to frequently induce feelings of boredom, frustration, anxiety, depression, and isolation among students, leading to distress and affecting their ability to cope [8].

A recent investigation into depressive symptoms among nursing students revealed that irritability was the most prevalent symptom. This was followed by uncontrollable worry, difficulty relaxing, a depressed mood, and anhedonia. These symptoms were identified as the most likely factors to initiate or perpetuate a state of depression among nursing students [9, 10]. Furthermore, a study by Kalkan et al. (2021) found that social isolation and emotional eating behaviors are common depressive symptoms among nursing students affected by the COVID-19 pandemic [11]. A study by Ullah and Amin in 2020 found that nursing students exhibit higher levels of depression compared to students from other disciplines [12]. This is attributed to the constant changes in clinical training settings, frequent interactions with new supervisors, and the need to familiarize themselves with different peer groups. Consequently, the coping mechanism employed by nursing students during this critical period is garnering the attention of global health organizations [1].

The coping mechanisms employed during routine life stressors may differ from those utilized during a health crisis, such as a pandemic. People are more likely to resort to maladaptive coping mechanisms during a crisis [13]. It has been observed that when faced with uncontrollable stressors, maladaptive coping mechanisms like avoidance can be more effective than problem-focused coping [14]. While an avoidant coping strategy allows for a gradual acknowledgment of the threat, it could exacerbate mental health issues in young people [15]. Conversely, initial evidence indicates that adaptive coping strategies, such as accepting the current situation and diligently practicing preventive measures like hand washing, wearing masks, and seeking accurate information about COVID-19, could help reduce symptoms of anxiety and depression [16, 17].

The transactional model of stress and coping developed by Lazarus and Folkman (1984) documented that stress results from the interaction between an individual and their environment, where the perception of a stressor and the appraisal of available coping resources play a significant role. The model suggests that individuals engage in cognitive appraisal processes to evaluate the demands of a situation and their ability to cope with it. Nursing students' coping mechanisms will be explored within this framework as they navigate the challenges and stressors associated with the COVID-19 pandemic [18].

The COVID-19 pandemic has presented unprecedented challenges and stressors for healthcare workers, including nursing students [19]. This study, through its examination of the prevalence of fear, coping strategies, and depressive symptoms, can shed light on the psychological impact experienced by nursing students during this crisis. It also offers valuable insights into the mental health needs of this group. Such information could aid healthcare institutions, policymakers, and educators formulate targeted interventions and support systems to address their unique challenges. Moreover, nursing students who display high levels of fear, depressive symptoms, and maladaptive coping could be at risk of delivering compromised patient care [20]. This underscores the urgency of addressing these psychological factors and providing the necessary support, as it directly impacts the professionalism and well-being of nursing students, ultimately leading to improved patient outcomes. Regrettably, there have been few studies examining depression among nursing students in Egypt and the MENA region during the COVID-19 pandemic, and their coping behaviors remain largely unknown [20, 21]. The current study examined the relationship between COVID-19 pandemic fear, coping strategies, and depressive symptoms among nursing students.

## Methods

### Study design

The study followed a cross-sectional survey that adhered to STROBE guidelines.

### Setting

The study was undertaken in the Faculty of Nursing at Alexandria University in Egypt. The faculty has nine scientific departments and offers academic degrees such as a Bachelor of Science in Nursing (BSC), postgraduate degrees including a nursing diploma, an MSC, and a Ph.D. in a specialized nursing branch. The faculty operates under the Ministry of Higher Education in Egypt and follows a credit hour system. The bachelor's degree program is eight semesters long and includes theoretical and clinical study, followed by a one-year internship in various healthcare facilities [22].

### Participants

The population for the current study consisted of undergraduate nursing students registered for the second term from 2019 to 2020. Records from the Student Affairs Department at the Faculty of Nursing showed that the total number of undergraduate students enrolled during this academic year was 1,910. These students were distributed across the four academic semesters as follows: 605 students were enrolled in the second semester of the first year, 382 in the fourth semester of the second year, 371 in the sixth semester of the third year, and 552 in the eighth semester of the fourth year.

### Eligibility criteria

The study included Egyptian undergraduate nursing students aged 18 years and older. The exclusion criteria encompassed non-Egyptian students, those who reported mental disorders or had a history of mental health issues, and students who were infected with the COVID-19 virus during the data collection period.

### Sample size calculation

The EPI INFO 7 program calculated the study's sample size. With an acceptable error of 5%, a confidence level of 95%, an expected frequency of 50%, and a population size of 1,910 undergraduate students, the program suggested that a minimum sample size of 319 students was required.

### Sampling and recruitment process

**Fig. 1:**
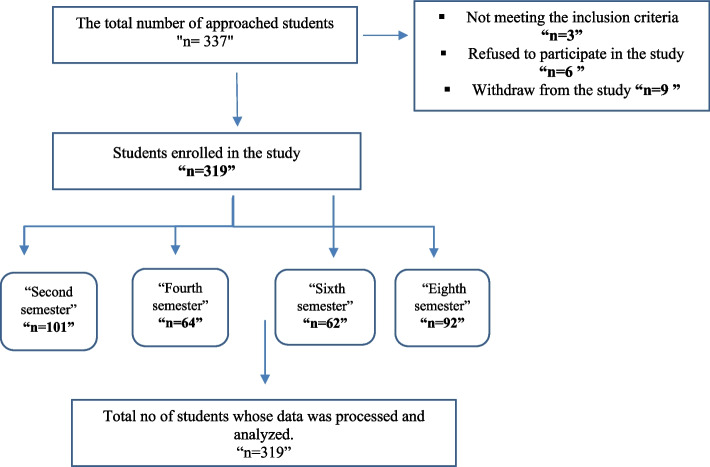
Flow chart of participants’ recruitment

The total number of students who were invited was 337. Of these, 18 students dropped out, with a response rate of 94.6%. As a result, a final sample of 319 students was chosen from four academic semesters using a stratified randomization technique and proportional allocation method to ensure a representative sample. The sample comprised 101 students from the 2nd semester, 64 from the 4th semester, 62 from the 6th semester, and 92 from the 8th semester (Fig. 1).

### Data collection tools

#### Fear of the coronavirus questionnaire (FCQ)

The FCQ was developed by Mertens, Gerritsen, et al. [23] during the COVID-19 pandemic [23]. It consists of eight items designed to measure the fear associated with COVID-19. Responses were given on a Likert-type scale ranging from 1 (Strongly Disagree) to 5 (Strongly Agree). The FCQ includes statements such as “I am very worried about the coronavirus” and “I am constantly following all news updates regarding the virus.” The total score can range from 8 to 40, with a score of 8–18 indicating mild fear, 19–29 suggesting moderate fear, and 30–40 signifying severe fear of the coronavirus. After the scale was translated into Arabic and back-translated, the researchers performed a factor analysis to assess its validity. The factor loadings ranged from 0.69 to 0.78 before and from 0.67 to 0.82 after rotation, exceeding the recommended threshold of 0.35 and accounting for 76.32% of the total variation. The Kaiser–Meyer–Olkin measure of sampling adequacy was 0.92, indicating adequate sampling. Furthermore, the statistically significant Bartlett's test of sphericity (*p* = 0.000) confirmed the correlation matrix's factorability and the scale items' appropriateness. The scale's internal consistency was acceptable, with a Cronbach's alpha coefficient of 0.77 for the full scale.

#### Brief coping orientation to problems experienced (COPE) scale

The COPE, developed by Carver (1997), is a standardized, self-reported questionnaire designed to assess an individual's coping behaviors and thoughts in response to stressful situations [24]. This widely used scale comprises 28 items and 14 coping strategies, with two items assigned to each. Respondents are asked to rate each of the 28 items on a four-point Likert scale, ranging from 1 ("I have not been doing this at all") to 4 ("I have been doing this a lot"). The coping mechanisms are categorized into two groups: adaptive and maladaptive. Adaptive coping mechanisms include active coping, positive reframing, planning, humor, acceptance, religion, and emotional and instrumental support. In contrast, maladaptive coping mechanisms encompass self-distraction, denial, substance use, behavioral disengagement, venting, and self-blame. The total score ranges from 28 to 112, with a higher score indicating more severe use of adaptive coping strategies. Scores from 28–56 reflect mild use, 57–84 indicate moderate use, and 85–112 suggest severe use. The standardized Arabic Version of the brief COPE tool was used in this study [25]. It demonstrated satisfactory internal reliability across all its subscales, with values ranging from 0.06 to 1.00.

#### Beck depression inventory (BDI-II)

The Beck Depression Inventory-II (BDI-II), developed by Beck et al. [26], is a self-report assessment of 21 items [26]. It is specifically designed to gauge the severity of depression in both adults and adolescents. The items in the BDI-II assess symptoms that align with the diagnostic criteria for depressive disorders as outlined in the Diagnostic and Statistical Manual of Mental Disorders. Participants rate the items on a 4-point Likert scale, with scores ranging from 0 (no symptoms) to 3 (severe symptoms). The total score on the BDI-II can range from 0 to 63, with higher scores indicating more severe depressive symptoms. The scoring categories for interpretation are as follows: a total score of 0–13 suggests mild mood disturbance, 14–19 is considered borderline clinical depression, 20–28 indicates moderate depression and 29–63 signifies severe depression. This study employed the standardized Arabic Version of the BDI-II, translated by Alansari [27]. The Arabic version demonstrated excellent internal consistency, with a Cronbach's alpha coefficient of 0.83 [27].

#### Sociodemographic and academic data profile

The researchers have developed a survey to gather information about different aspects. This includes basic details such as gender, marital status, living situation, and academic semester. Moreover, it contains questions that aim to comprehend the students' enthusiasm and drive to pursue nursing sciences, along with their work experiences during their academic years.

### Procedure

#### Ethical approval

The research study obtained ethical approval from the Research Ethical Committee (REC) (IRB00013622/813/1/2020) at the Faculty of Nursing, Alexandria University. All students were given a comprehensive explanation of the study's objectives and detailed information about the questionnaire via their academic emails. Before distributing the questionnaire, electronic consent was secured from each participant, ensuring they understood the study's purpose and voluntarily agreed to participate. Participants were reassured that their information would remain confidential. They were also explicitly informed of their right to refuse participation or withdraw from the study at any point without any adverse consequences. No financial incentives were offered to participants for their involvement in the study.

#### Pilot study

A pilot study involved 40 randomly chosen undergraduate students to pinpoint any challenges during the data collection. The pilot study confirmed that the tools were accurate, understandable, and suitable.

#### Data collection

The researchers procured a list of registered students' names, academic semesters, and emails from the Faculty of Nursing's affairs department for the four academic semesters of the 2019–2020 "second-term" academic year. A representative sample of students was selected using a stratified random sampling method, excluding those who participated in the pilot study and the reliability test. The following steps were taken: all registered students in the four semesters (2nd, 4th, 6th, and 8th semesters) were divided into four strata, and a representative sample of students from each stratum (semester) was selected using a simple randomization technique and the proportional allocation method. The students were given a link to a survey containing the study's objectives and an informed consent form. The questionnaire was only accessible after agreeing to the consent form. Additionally, the privacy and confidentiality of their responses were ensured. The instructions on how to respond to the survey were also provided. The data collection process occurred between April and August 2020.

#### Data analysis and processing

The data collected from Microsoft Form sheets were analyzed using the Statistical Package for Social Sciences (SPSS) 25.0 V program. The reliability of the tools was evaluated using Cronbach's alpha test. The Shapiro–Wilk (SW) and Kolmogorov–Smirnov (KS) tests ensured the data's normality. The correlation between two quantitative variables was measured using the Pearson coefficient, and significant levels were determined at *p*-values of 0.001 and 0.05. A multiple regression analysis model was employed to determine the degree of variance in depressive symptoms and coping mechanisms due to coronavirus fear as an independent variable.

## Results

### The demographic and academic characteristics of the participants

Table 1 displays that most participants were female (70.8%). The largest age group (57.1%) was between 20 to 24 years old. Nearly all participants were single (97.8%), lived in cities (90%), and lived with family members (93.1%). Regarding academic grades, 31.6% were in their first year, 20.2% in their second, 19.4% in their third, and 28.8% in their fourth academic year. The table also shows that 44.5% of participants joined the Faculty of Nursing based on their grades, while 28.5% joined because they wanted to serve others. Additionally, 19.7% of participants worked in private hospitals while studying, while the majority (80.3%) did not work while studying.

**Table 1:** The Participants sociodemographic and academic Characters *(n* = *319)*

Variables		Total	
		**n**	**%**
**Gender**	Male	93	29.2
	Female	226	70.8
**Age**	≥ 20	122	38.2
	20- 24	182	57.1
	≤ 25	15	4.7
**Grade**	First Year	101	31.6
	Second Year	64	20.2
	Third Year	62	19.4
	Fourth Year	92	28.8
**Marital status**	Single	312	97.8
	Married	7	2.2
**Working during studying years**	Yes	63	19.7
	No	256	80.3
**Reason for joining the faculty of nursing**	Total Marks/The Educational System	142	44.5
	Getting a Job	75	23.5
	Wanting to help other people	91	28.5
	Parents' wish	11	3.4
**Residence**	Rural	32	10.0
	Urban	287	90.0
**Cohabitation**	With Family	297	93.1
	Campus	10	3.1
	With Relatives	8	2.5
	Alone	4	1.3

### The prevalence of the coronavirus fear, coping strategies, and depressive symptoms among the participants

Table 2 shows that the average FCQ score was 28.92 (SD = 5.10). More than half of the participants (52.0%) reported having moderate fear, while 45.5% reported severe fear. The table also shows that the participants utilized coping mechanisms at a moderate level (64.6%) with a mean COPE score of 61.29 (SD = 10.58), while 34.5% utilized coping mechanisms at a mild level. In terms of their responses to the BDI-II, 41.4% of the participants reported mild depressive symptoms, whereas an equal number of participants (21.0% and 21.9%, respectively) reported moderate or severe depressive symptoms.

**Table 2:** Distribution of the studied candidates on FCQ, COPE, and BDI-II (*n* = 319)

Variables		Total	
		**n**	**%**
*FCQ	Mild fear	8	2.5
	Moderate fear	166	52.0
	Severe fear	145	45.5
	Mean (SD)	28.92 (5.10)	
**COPE	Mild use	110	34.5
	Moderate use	206	64.6
	Severe use	3	0.9
	Mean (SD)	61.29 (10.58)	
***BDI-II	Mild depression	132	41.4
	Borderline depression	50	15.7
	Moderate depression	67	21.0
	Severe depression	70	21.9
	Mean (SD)	20.14 (14.29)	

**FCQ:** Fear of the Coronavirus Questionnaire, **COPE:** Brief Coping Orientation to Problems Experienced Scale, **BDI-II**: Beck Depression Inventory

^*^Mild (8-18), Moderate1(9-29), Severe (30-40)

^**^Mild (28–56), Moderate (57–84), Severe (85–112)

^***^Mild (0-13), Borderline (14-19), Moderate (20-28), Severe (29-63)

### Coping mechanisms used by the participants

**Fig. 2:**
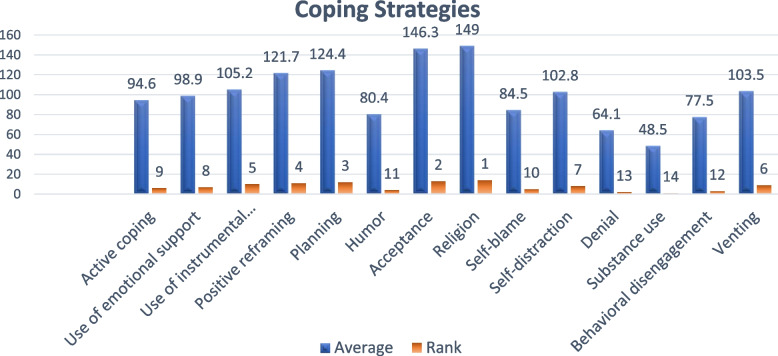
Distribution of adaptive and maladaptive coping strategies on the COPE scale among the participants (*n* = 319)

Figure 2 demonstrates that among the participants, the coping mechanisms of "religion" and "acceptance" were the most commonly utilized, with percentages of 149% and 146.3% respectively. This was followed by "planning" and "positive reframing," with percentages of 124.4% and 121.7%, respectively. The coping mechanisms of "use of instrumental support" and "venting" were also employed. Additionally, after the "self-distraction approach," the students applied “emotional support” and “active coping” strategies. The least frequently used coping mechanisms by the students were “self-blame,” “humor,” “behavioral disengagement,” “denial,” and “substance abuse,” with percentages of 84.5%, 80.4%,77.5%, 64.1%, and 48.5%, respectively.

### The correlation coefficient between the studied variables

Table 3 reveals a statistically significant positive correlation between fear of the coronavirus and adaptive (*r* = 0.376, *p* = 0.01) and maladaptive (*r* = 0.168, *p* = 0.01) coping strategies. A statistically significant correlation exists between fear of the coronavirus and depression (*r* = 0.160, *p* = 0.01). Furthermore, the table shows a significant positive correlation between depression and maladaptive coping (*r* = 0.352, *p* = 0.01). Meanwhile, a significant negative correlation was found between depression and adaptive coping mechanisms (*r* = -0.074, *p* = 0.01).

**Table 3:** The correlation coefficients Matrix between FCQ, COPE and BDI-II among the participants

Variables	Coronavirus fear (FCQ)	Coping strategies		Depressive symptoms (BDI-II)
		**Adaptive coping**	**Maladaptive coping**	
**FCQ**				
**Adaptive coping**	.376**			
**Maladaptive coping**	.168**	.374**		
**BDI-II**	.160**	-.074	.352**	

*FCQ***:** Fear of the Coronavirus Questionnaire, *COPE***:** Brief Coping Orientation to Problems Experienced Scale, *BDI-II*: Beck Depression Inventory

r = Pearson correlation ** Statistically significant value at P ≤ 0.01

r ≥ 0.9 very high correlation r 0.7- < 0.9 high correlation r 0.5- < 0.7 moderate correlation r < 0.5 low correlation

### The multivariate regression analysis

Table 4 presents a multivariate regression analysis that examined the relationships between the dependent variable (depressive symptoms) and the independent variables (fear, adaptive, and maladaptive coping strategies). In the depressive symptoms model, fear had an unstandardized coefficient of 0.379, suggesting that for each unit increase in fear, there was a corresponding increase of 0.379 in depressive symptoms. The standardized coefficient (Beta) for fear was 0.135, indicating that fear accounted for approximately 13.5% of the variance in depressive symptoms. The constant term was 9.171, representing the expected value of depressive symptoms when all independent variables were zero.

**Table 4:** Multivariate linear regression model between coronavirus fear, coping strategies and depression among the studied candidates (*n* = 319)

Dependent variable	Model	Unstandardized coefficients		Standardized coefficients	t	F	R square
		**B**	**Std. Error**	**Beta**			
**Depressive symptoms**	**(Constant)**	9.171	4.549	––	2.016*	5.991*	1.8%
	**Fear**	0.379	0.155	0.135	2.448*		
**Adaptive coping strategies**	**(Constant)**	23.382	2.347	––	9.961**	53.094**	14.2%
	**Fear**	0.583	0.080	0.376	7.287**		
**Maladaptive coping strategies**	**(Constant)**	16.528	1.490	––	11.095**	9.341**	2.8%
	**Fear**	0.155	0.051	0.168	3.056**		

R2: Coefficient of determination. F, p: f, and p values for the model

B: Unstandardized Coefficients. Beta: Standardized Coefficients

t: t-test of significance. *: Statistically significant value at p ≤ 0.05. **: Statistically significant value at *p* ≤ 0.01

In the adaptive coping mechanism model, fear had an unstandardized coefficient of 0.583, indicating that for each unit increase in fear, depressive symptoms increased by 0.583. The standardized coefficient (Beta) for fear was 0.376, suggesting that fear explained approximately 37.6% of the variance in depressive symptoms. The constant term was 23.382, representing the expected value of depressive symptoms when all independent variables were zero.

In the maladaptive coping mechanism model, fear had an unstandardized coefficient of 0.155, indicating that for each unit increase in fear, depressive symptoms increased by 0.155. The standardized coefficient (Beta) for fear was 0.168, suggesting that fear explained approximately 16.8% of the variance in depressive symptoms. The constant term was 16.528, representing the expected value of depressive symptoms when all independent variables were zero.

Overall, fear was positively associated with depressive symptoms. However, when adaptive coping mechanisms were employed, fear showed a negative association with depressive symptoms, indicating that higher levels of adaptive coping were associated with lower levels of depressive symptoms. Conversely, in the presence of maladaptive coping strategies, fear was positively associated with depressive symptoms, suggesting that higher levels of fear were linked to higher levels of depressive symptoms. These findings highlight the importance of considering coping mechanisms when understanding the relationship between fear and depressive symptoms.

## Discussion

Nursing is a profession that plays a pivotal role in patient care during any pandemic. The widespread disruption of daily life, uncertainty, and fear caused by the novel coronavirus (COVID-19) pandemic has had a significant psychological impact [27]. Depressive symptoms are mental health issues that can affect anyone, including nursing students. It is well-known that nurses are at the forefront of the battle against the COVID-19 pandemic. In this context, the current study explored the relationship between fear of the coronavirus, coping strategies, and depressive symptoms among nursing students. Our results were interpreted in light of previous and similar literature that estimates the prevalence of mental health issues among healthcare workers [28–33].

Our study revealed that a significant portion of the participants exhibited a moderate fear related to the coronavirus. These findings align with the global understanding that the current health crisis has a significant negative impact on the psychological well-being of college students. In this context, a study by Odriozola-González et al. [34] confirmed that academic students reported significantly higher scores for fear and stress [35]. The present study's findings highlight the fear experienced by nursing students during this stressful period, which could be attributed to the communicability and lethal nature of the new coronavirus pandemic, which has high incidence and mortality rates. Students' fear might also be centered around the possibility of their family members, friends, and colleagues becoming infected. Disrupting their daily activities and reduced social contact and support might also contribute to their fear.

Another significant finding of this study is that 21.9% of the students surveyed exhibited severe depressive symptoms. These results align with other studies conducted in Arab countries, which reported that participants exhibited the highest levels of depression during the COVID-19 crisis [18, 32, 34]. Additionally, the findings of Al-Sabbah et al. [36] suggested that responses to statements such as “raising the degree of depression”, “being less happy”, and “having negative thoughts” could be interpreted as adverse effects of the pandemic on the Egyptian population [37]. This could be interpreted from a biological standpoint. The enforced quarantine and reduced outdoor activities could lead to insufficient sunlight exposure, decreasing serotonin levels. This decrease is associated with emotional disorders such as anxiety and depression [36]. The restrictions on social interactions with family members, friends, and colleagues could induce helplessness, hopelessness, and uncertainty. Disruptions to daily activities may also contribute to psychological issues related to stress and depression [38–40]. During their learning experiences, students faced direct and indirect exposure to numerous risk factors, such as interacting with infected patients or handling contaminated surfaces in various clinical settings. Another significant event during this period was the sudden transition from traditional face-to-face educational methods to online classes. This shift imposed a considerable psychological and financial burden on the students, triggering fear and depressive symptoms. Moreover, the excessive use of technological devices like mobile phones, computers, and laptops for their studies can increase stress and depression [41]. The vast amount of information they receive from various sources, including social media, could contain myths and misinformation about the pandemic, potentially triggering feelings of stress, anxiety, and depressive symptoms.

The current findings reveal that a significant portion of the participants used moderate coping mechanisms. This aligns with the findings of Al-Shannaq et al. (2021), who examined depression, coping skills, and quality of life among Jordanian adults during the initial outbreak of the COVID-19 pandemic and found that participants had a moderate ability to cope with their situation effectively [28]. This can be related to the fact that the use of specific coping Mechanisms during a threat may be influenced by students' perceptions of the severity of the viral infection and their beliefs, in addition to their views on the importance of preventive measures or the use of health and medical services [42]. This is also in line with previous studies, which found that most subjects studied exhibited more adaptive health behaviors and higher compliance rates with official recommendations [42–44].

The study discovered a correlation between fear of the coronavirus, coping strategies, and symptoms of depression. It was found that fear of the coronavirus increased students' adaptive coping by 14.2%. This aligns with the findings of Al-Shannaq et al. (2021), who identified a connection between depression and coping skills during the initial outbreak of the COVID-19 pandemic [28]. However, this contrasts with the results of Skapinakis et al. [43], who showed that positive coping behaviors related to the coronavirus were negatively associated with depression. In contrast, more dysfunctional methods such as denial, drug use, and surrender were more strongly linked [42].

According to our findings, students with symptoms of depression may have employed coping mechanisms to manage their depression during the COVID-19 pandemic. These findings could be attributed to individuals resorting to adaptive and maladaptive coping mechanisms to handle perceived threats during stressful situations. For example, students experiencing intense fear might resort to avoidance, denial, and substance use as coping strategies. While these methods might alleviate immediate distress, they could lead to feelings of isolation and symptoms of depression. Maladaptive coping mechanisms such as denial, self-destruction, substance abuse, venting, self-blame, and behavioral disengagement might temporarily distort a student's perception of reality. Consequently, uncertainty and unpredictability might emerge, potentially leading to helplessness and hopelessness [17, 20].

### Limitations of the study

Although the study provided valuable insights, it had several limitations that may need to be revised to allow the generalizability of the results. The use of self-reported questionnaires on fear, depression, and coping during the COVID-19 pandemic may also have some limitations. Self-reported measures may be subject to bias. The accuracy of the responses may be affected by factors such as social desirability bias, recall bias, and response bias. Furthermore, online surveys may introduce selection bias, as participants who are more comfortable with technology may be more likely to participate. Future studies may consider using longitudinal or qualitative methods to overcome these limitations. Longitudinal studies can help track mental health changes over time. On the other hand, qualitative studies can provide a more in-depth understanding of individuals' experiences and perspectives during the pandemic. Objective measures such as physiological responses or behavioral observations may also provide more accurate data on fear and coping.

## Conclusion

During the COVID-19 pandemic, nursing students experienced intense fear and depressive symptoms while employing moderate coping mechanisms. The study identified a significant correlation between the fear of COVID-19 and depressive symptoms. However, this correlation is modulated by the type of coping strategy used. When effectively employed, adaptive coping strategies can lessen the impact of fear on depressive symptoms. On the other hand, maladaptive coping strategies can intensify the positive link between fear and depressive symptoms. Thus, the study highlights the crucial role of fostering adaptive coping strategies in managing the psychological repercussions of the COVID-19 pandemic. Nursing faculties should provide accurate information about COVID-19 and offer access to trained counselors for student support. Tele-mental health interventions and relaxation techniques can be implemented to help students cope with the pandemic's psychological impact. Teaching adaptive coping strategies and highlighting the risks of maladaptive behaviors is crucial. Online platforms can be utilized for educational resources and video conferences.

## Data Availability

The datasets used and analyzed during the current study are available from the corresponding author upon reasonable request.

## Abbreviations

WHO: World Health Organization
BDI-II: Beck Depression Inventory
FCQ: Fear of the Coronavirus Questionnaire
COPE: Brief Coping Orientation to Problems Experienced
SW: Shapiro–Wilk
KS: Kolmogorov–Smirnov
SPSS: Statistical Package for Social Sciences
REC: Research Ethical Committee

## References

[1] WHO. Coronavirus disease (COVID-19) pandemic. 2020 https://www.who.int/europe/emergencies/situations/covid-19

[2] Holmes EA, O’Connor RC, Perry VH, Tracey I, Wessely S, Arseneault L (2020). Multidisciplinary research priorities for the COVID-19 pandemic: a call for action for mental health science. Lancet Psychiatry.

[3] Pfefferbaum B, North CS. Mental health and the Covid-19 pandemic. New England journal of medicine. 2020;383(6):510–2. https://www.nejm.org/doi/10.1056/NEJMp2008017 10.1056/NEJMp2008017 32283003

[4] Qiu JY, Zhou DS, Liu J, Yuan TF (2020). Mental wellness system for COVID-19. Brain Behav Immun.

[5] Al-Rabiaah A, Temsah MH, Al-Eyadhy AA, Hasan GM, Al-Zamil F, Al-Subaie S (2020). Middle East Respiratory Syndrome-Corona Virus (MERS-CoV) associated stress among medical students at a university teaching hospital in Saudi Arabia. J Infect Public Health.

[6] Sahu P. Closure of universities due to coronavirus disease 2019 (COVID-19): impact on education and mental health of students and academic staff. Cureus. 2020;12(4). https://www.ncbi.nlm.nih.gov/pmc/articles/PMC7198094/10.7759/cureus.7541 PMC7198094 32377489

[7] Kecojevic A, Basch CH, Sullivan M, Davi NK (2020). The impact of the COVID-19 epidemic on the mental health of undergraduate students in New Jersey, cross-sectional study. PLoS ONE.

[8] Chang J, Yuan Y, Wang D. Mental health status and its influencing factors among college students during the epidemic of COVID-19. Nan Fang Yi Ke Da Xue Xue Bao. 2020;171–6. https://www.j-smu.com/CN/10.12122/j.issn.1673-4254.2020.02.06 10.12122/j.issn.1673-4254.2020.02.06 PMC7086131 32376528

[9] Bai W, Xi HT, Zhu Q, Ji M, Zhang H, Yang BX (2021). Network analysis of anxiety and depressive symptoms among nursing students during the COVID-19 pandemic. J Affect Disord.

[10] Abdelbaky Ahmed E, Mohamed Mourad G, Sayed Mohamed H, Mohamed Hassan S. Perceived stressors and coping patterns among nursing students during Covid-19 Pandemic. Egypt J Health Care. 2023 Dec 1;14(4):283–95. 10.21608/ejhc.2023.326601

[11] Kalkan Uğurlu Y, Mataracı Değirmenci D, Durgun H, Gök UH (2021). The examination of the relationship between nursing students' depression, anxiety, and stress levels and restrictive, emotional, and external eating behaviors in the COVID-19 social isolation process. Perspective Psychiatry Care.

[12] Ullah R, Amin S (2020). The psychological impact of COVID-19 on medical students. Psychiatry Res.

[13] Kamaludin K, Chinna K, Sundarasen S, Khoshaim HB, Nurunnabi M, Baloch GM, et al. Coping with COVID-19 and movement control order (MCO): experiences of university students in Malaysia. Heliyon. 2020;6(11). 10.1016/j.heliyon.2020.e05339 10.1016/j.heliyon.2020.e05339 PMC7584419 33134570

[14] Main A, Zhou Q, Ma Y, Luecken LJ, Liu X (2011). Relations of SARS-related stressors and coping to Chinese college students’ psychological adjustment during the 2003 Beijing SARS epidemic. J Couns Psychol.

[15] Liang L, Ren H, Cao R, Hu Y, Qin Z, Li C, et al. The effect of COVID-19 on youth mental health. Psychiatric quarterly. 2020; 91:841–52. https://link.springer.com /article/10.1007/s11126-020-09744-3 10.1007/s11126-020-09744-3 PMC7173777 32319041

[16] Wang C, Pan R, Wan X, Tan Y, Xu L, Ho CS (2020). Immediate psychological responses, and associated factors during the initial stage of the 2019 coronavirus disease (COVID-19) epidemic among the general population in China. Int J Environ Res Public Health.

[17] Omar DI, Amer SA, Abdelmaksoud AE. Fear of COVID-19, Stress and Coping Strategies among Nurses during the COVID-19 Pandemic’s Second Wave: A Quasi-Intervention Study. Clinical Practice and Epidemiology in Mental Health: CP & EMH. 2023;19. 10.2174/18740179-v18-e221221-2022-2 PMC10156021 38130816

[18] Lazarus RS, Folkman S. Stress, appraisal, and coping. Berlin: Springer Publishing Company;1984.

[19] Alsolais A, Alquwez N, Alotaibi KA, Alqarni AS, Almalki M, Alsolami F (2021). Risk perceptions, fear, depression, anxiety, stress and coping among Saudi nursing students during the COVID-19 pandemic. J Ment Health.

[20] Shaban IA, Khater WA, Akhu-Zaheya LM (2012). Undergraduate nursing students' stress sources and coping behaviors during their initial clinical training period: A Jordanian perspective. Nurse Educ Pract.

[21] Wang R, Zhou C, Wu Y, Sun M, Yang L, Ye X (2022). Patient empowerment and self-management behavior of chronic disease patients: A moderated mediation model of self-efficacy and health locus of control. J Adv Nurs.

[22] Alexandria University. Faculty of Nursing. Retrieved on May 2022. Available at: https://nurs.alexu.edu.eg/index.php/en/

[23] Mertens G, Gerritsen L, Duijndam S, Salemink E, Engelhard IM (2020). Fear of the coronavirus (COVID-19): Predictors in an online study conducted in March 2020. J Anxiety Disorder.

[24] Carver CS. You want to measure coping, but your protocol is too long: Consider the brief cope. Int J Behav Med. 1997;4(1):92–100. https://link.springer.com/article/10.1207/s15327558ijbm0401_6 10.1207/s15327558ijbm0401_6 16250744

[25] Alghamdi M (2020). Cross-cultural validation and psychometric properties of the Arabic Brief COPE in Saudi population. Med J Malaysia.

[26] Beck AT, Steer RA, Brown G. Beck depression inventory–II. Psychol Assess. 1996. https://psycnet.apa.org/doi/10.1037/t00742-000

[27] Alansari BM (2006). Internal consistency of an Arabic adaptation of the Beck Depression Inventory-II with college students in eighteen Arab countries. Soc Behav Personal Int J.

[28] Sugawara D, Chishima Y, Kubo T, Reza RIABR, Phoo EYM, Ng SL, et al. Mental health and psychological resilience during the COVID-19 pandemic: A cross-cultural comparison of Japan, Malaysia, China, and the US. J Affect Disord. 2022; 311:500–7. 10.1016/j.jad.2022.05.032 10.1016/j.jad.2022.05.032 PMC9090817 35561884

[29] Al-Shannaq Y, Mohammad AA, Aldalaykeh M. Depression, coping skills, and quality of life among Jordanian adults during the initial outbreak of COVID-19 pandemic: cross-sectional study. Heliyon. 2021;7(4). 10.1016/j.heliyon.2021.e06873 10.1016/j.heliyon.2021.e06873 PMC8095109 33997404

[30] Al Omari O, Al Sabei S, Al Rawajfah O, Abu Sharour L, Aljohani K, Alomari K (2020). Prevalence and predictors of depression, anxiety, and stress among youth at the time of COVID-19: an online cross-sectional multicounty study. Depress Res Treat.

[31] Alsharji KE. Anxiety and depression during the COVID-19 pandemic in Kuwait: the importance of physical activity. Middle East Current Psychiatry. 2020;27(1):1–8. https://mecp.springeropen.com/articles/10.1186/s43045-020-00065-6

[32] El-Monshed AH, Loutfy A, Saad MT, Ali AS, El-Gilany AH, Soliman Mohamed A (2022). Satisfaction with life and psychological distress during the COVID-19 pandemic: An Egyptian online cross-sectional study. Afr J Prim Health Care Fam Med.

[33] Megreya  AM,  Latzman RD, Al-Ahmadi AM, Al-Dosari NF (2022). he COVID-19-related lockdown in Qatar: Associations among demographics, social distancing, mood changes, and quality of life. Int J Ment Health Addict.

[34] Odriozola-González P, Planchuelo-Gómez Á, Irurtia MJ, de Luis-García R (2020). Psychological effects of the COVID-19 outbreak and lockdown among students and workers of a Spanish university. Psychiatry Res.

[35] Thomas J, Barbato M (2020). Positive religious coping and mental health among Christians and Muslims in response to the COVID-19 pandemic. Religions (Basel).

[36] Al-Sabbah S, Darwish A, Fares N, Barnes J, Almomani JA (2021). Biopsychosocial factors linked with the overall well-being of students and educators during the COVID-19 pandemic. Cogent Psychol.

[37] Shuwiekh HAM, Kira IA, Sous MSF, Ashby JS, Alhuwailah A, Baali SBA, et al. The differential mental health impact of COVID-19 in Arab countries. Current Psychology. 2020;1–15. https://link.springer.com/article/10.1007/s12144-020-01148-7 10.1007/s12144-020-01148-7 PMC7605480 33162726

[38] Cao W, Fang Z, Hou G, Han M, Xu X, Dong J (2020). The psychological impact of the COVID-19 epidemic on college students in China. Psychiatry Res.

[39] Ellis JR, Hartley CL. Nursing in today’s world: trends, issues & management. Lippincott Williams & Wilkins; 2004. https://cmc.marmot.org/Record/.b41446045

[40] Tian-Ci Quek T,  Wai-San Tam W, X. Tran B, Zhang M, Zhang Z, Su-Hui Ho C (2019). The global prevalence of anxiety among medical students: a meta-analysis. Int J Environ Res Public Health.

[41] Trivate T, Dennis AA, Sholl S, Wilkinson T (2019). Learning and coping through reflection: exploring patient death experiences of medical students. BMC Med Educ.

[42] Dhawan S (2020). Online learning: A panacea during the COVID-19 crisis. J Educ Technol Syst.

[43] Skapinakis P, Bellos S, Oikonomou A, Dimitriadis G, Gkikas P, Perdikari E (2020). Depression and its relationship with coping strategies and illness perceptions during the COVID-19 lockdown in Greece: a cross-sectional survey of the population. Depress Res Treat.

[44] Chew QH, Wei KC,  Vasoo S, Chua HC,  Sim K (2020). Narrative synthesis of psychological and coping responses towards emerging infectious disease outbreaks in the general population: practical considerations for the COVID-19 pandemic. Singapore Med.

